# Entrepreneurial calling and psychological wellbeing: a qualitative study of a dynamic and regulated work experience

**DOI:** 10.3389/fpsyg.2026.1830938

**Published:** 2026-06-24

**Authors:** Judit Jakab, Attila Oláh

**Affiliations:** 1 Doctoral School of Psychology, Faculty of Education and Psychology, Eötvös Loránd University, Budapest, Hungary; 2 Department of Personality and Health Psychology, Faculty of Education and Psychology, Institute of Psychology, Eötvös Loránd University, Budapest, Hungary

**Keywords:** autonomy, entrepreneurial calling, identity, meaningful work, psychological wellbeing, thematic analysis

## Abstract

**Introduction:**

Calling is commonly associated with meaningful, identity-defining work and positive psychological outcomes. Although entrepreneurial work is often characterized by strong personal engagement and meaningful self-directed activity, less is known about how entrepreneurial calling is experienced in everyday work practice and how it relates to psychological wellbeing.

**Methods:**

This study draws on in-depth, semi-structured interviews with 31 Hungarian entrepreneurs who perceived their work as a calling. Thematic analysis explored how participants experienced, enacted, and sustained entrepreneurial calling in everyday work practice, including the meaning of their work, identity-relevant engagement, and the psychological consequences of living a calling within entrepreneurial contexts.

**Results:**

Participants experienced calling through meaningful work activities, with entrepreneurship functioning as a flexible context enabling identity-relevant and self-directed work. Entrepreneurial calling emerged as a dynamic form of engagement characterized by: (1) identity-congruent meaning and intrinsic engagement; (2) autonomy-enabled self-direction; (3) uncertainty, emotional strain, and blurred boundaries; and (4) growth, self-reflection, adaptation, and boundary negotiation. Psychological wellbeing did not emerge automatically from calling, but depended on participants’ capacity to sustain engagement while managing its emotional and personal demands over time.

**Discussion:**

Findings suggest that entrepreneurial calling represents a dynamically enacted form of meaningful work engagement shaped through the interplay between identity, autonomy, uncertainty, and psychological demand. While identity-congruent and autonomy-enabled work fostered meaning, purpose, and personal growth, it also intensified emotional strain and difficulties disengaging from work. Entrepreneurial calling therefore emerged as an evolving and psychologically complex process requiring continuous adaptation and negotiation to remain sustainable.

## 1 Introduction

The idea of work as a calling has attracted sustained interest in psychological and organizational research (Wrzesniewski et al., 1997; Dik and Duffy, 2009). Calling is commonly understood as a deeply felt sense of purpose that draws individuals toward a particular form of work experienced as personally meaningful, intrinsically motivating, and socially valuable (Dik et al., 2012), and is often perceived as integral to one’s identity and life meaning (Bunderson and Thompson, 2009). In this sense, it is distinct from and more complex than passion, which is commonly understood as a strong affective attachment toward an activity (Vallerand et al., 2003; Cardon et al., 2009). Calling, in contrast, involves meaning, identity integration, and a broader sense of purpose that may be absent from passion-oriented engagement (Dik and Duffy, 2009; Bunderson and Thompson, 2009). This distinction is particularly important in entrepreneurial contexts, where work may become closely intertwined with personal identity and psychological wellbeing. Importantly, the present study does not examine calling within traditionally institutionalized or socially recognized “calling professions” such as medicine, teaching, or religious work, which have frequently shaped prior calling research (e.g., Bloom et al., 2021). Instead, we examine how calling is experienced and enacted within entrepreneurial contexts, where work is often self-created, less institutionally structured, and more directly shaped by individual autonomy, identity investment, and personal responsibility.

Prior research suggests calling may be experienced in diverse contexts, depending on how work activities align with identity, values, and purpose, rather than formal employment status alone (Dik and Duffy, 2009; Wrzesniewski and Dutton, 2001). It is often associated with positive outcomes such as job satisfaction, intrinsic motivation, and psychological wellbeing (Dobrow and Tosti-Kharas, 2011; Duffy et al., 2013). Yet when work becomes an expression of the self rather than a role performed (Bunderson and Thompson, 2009), this may heighten vulnerability to exhaustion and burnout, difficulties in disengaging, particularly when boundaries between professional and personal life are blurred (Duffy and Dik, 2013; Duffy et al., 2018).

Recent integrative reviews have emphasized that work experienced as a calling is inherently ambivalent, involving not only moral commitment, identity investment, and meaning but also significant personal and psychological costs (Thompson and Bunderson, 2019).

In entrepreneurial contexts, calling may take on a particularly intensive and personally consequential form because such work often combines high autonomy, strong identity investment, uncertain outcomes, and direct personal responsibility for success and failure (Murnieks et al., 2020; Stephan, 2018). These dynamics may become intensified through financial uncertainty, work overload, and personal responsibility for outcomes (Hessels et al., 2018; Shepherd et al., 2016). Recent work also suggests the role of individual and contextual resources, such as mental endurance, physical wellbeing, and institutional support, in shaping entrepreneurial engagement and aspirations (Sarwar et al., 2025).

Entrepreneurial calling provides a particularly important context for examining how meaningful and identity-relevant work is enacted and sustained under conditions of uncertainty and self-direction (Stephan, 2018). Entrepreneurial work often combines autonomy, intrinsic motivation, strong identity investment, and personal responsibility for outcomes (Murnieks et al., 2020), while simultaneously involving persistent uncertainty, stress, and the need for ongoing coping and resilience (Ahmed et al., 2022; Stephan, 2018). Previous research has also highlighted the role of personal meaning and self-direction in shaping entrepreneurial engagement (Baum and Locke, 2004; Cardon and Kirk, 2015). Studying calling in such contexts may therefore reveal how meaningful work is psychologically negotiated and sustained within highly self-directed forms of work engagement.

From a eudaimonic perspective on wellbeing, entrepreneurial calling is especially relevant for examining how key dimensions of psychological wellbeing, including meaning, autonomy, and personal growth, are experienced and negotiated within demanding forms of work engagement (Shir and Ryff, 2021). Contemporary work has advanced a more integrative view of wellbeing that acknowledges ambivalence, struggle, and the role of adversity in meaningful living. Rather than focusing primarily on positive affect or life satisfaction, these approaches emphasize eudaimonic dimensions such as purpose, authenticity, and personal growth, and conceptualize wellbeing as an ongoing process shaped through engagement with challenging life conditions (Lomas and Ivtzan, 2016; Wong et al., 2022). This is particularly relevant for studying entrepreneurial calling because it allows us to examine how meaningful and identity-relevant work may simultaneously involve fulfilment, psychological strain, and continuous self-regulation. By bringing eudaimonic approaches to wellbeing into conversation with research on entrepreneurial calling, we are better able to examine how entrepreneurs negotiate the interrelations between meaning, identity, psychological demands, and wellbeing in everyday work contexts.

Despite the growing literature on calling, entrepreneurship, and wellbeing, previous studies have predominantly relied on quantitative designs and employee-based samples, leaving limited understanding of how calling is experienced and sustained in everyday entrepreneurial work practice (Dobrow et al., 2023; Hart and Hart, 2023). Qualitative and narrative-oriented studies have begun to conceptualize calling as an evolving process of identity construction and meaning-making. Bloom et al. (2021), for example, showed how professionals in traditionally recognized calling-oriented occupations construct narrative identities through processes of discernment, authenticity, and professional legitimacy. Extending this process-oriented perspective into entrepreneurial contexts, Schou (2022) conceptualized entrepreneurial calling as an ongoing narrative and identity-related process. However, less attention has been paid to how entrepreneurial calling is psychologically sustained and negotiated in everyday work practice under conditions of autonomy, uncertainty, and ongoing psychological demand. Similarly, entrepreneurship research has rarely examined how meaning, psychological strain, and wellbeing are dynamically negotiated within entrepreneurial work contexts (Stephan, 2018; Shir and Ryff, 2021).

Drawing on this process-oriented perspective and Schou’s (2022) idea that entrepreneurial calling can be understood as a narrative process of making sense of work and identity over time, we define it as a form of calling in which individuals experience self-directed or self-created work as deeply meaningful, identity-congruent, and intrinsically motivating. We focus on how calling is experienced in relation to specific work activities enacted within entrepreneurial settings in order to understand how entrepreneurship may uniquely intensify the experience of calling by combining autonomy, identity investment, and direct responsibility for outcomes.

The present study therefore explores how entrepreneurs who perceive their work as a calling experience and negotiate meaning, identity, and psychological wellbeing in everyday work practice. Drawing on 31 in-depth interviews and thematic analysis, the study examines how entrepreneurial calling is enacted, negotiated, and sustained under conditions of autonomy, uncertainty, and ongoing psychological demand.

## 2 Methods

### 2.1 Research design

This study adopted an exploratory qualitative research design informed by a contextualist epistemological stance. We approached participants’ experiences of entrepreneurial calling and wellbeing as psychologically real and meaningful, while recognizing that these are always expressed through personal interpretation and shaped by broader social and professional contexts.

From a critical realist perspective, we assume that phenomena such as autonomy, strain, identity development, or emotional wellbeing are not merely linguistic constructions, but reflect lived psychological realities. At the same time, we acknowledge that such realities are accessed through participants’ subjective interpretations and narratives, shaped by personal, social, and contextual influences. This allows us to move beyond purely descriptive accounts and explore underlying patterns and mechanisms in how calling is experienced. Our aim was therefore not to establish universal truths about entrepreneurial calling, but to identify recurring patterns in how participants understand and experience it, particularly in relation to psychological wellbeing, tension, and sustainability over time. This position informed both data collection and analysis, supporting an inductive thematic approach that remained grounded in lived experience while allowing for interpretive engagement with the data, and guiding the analytic focus on identifying not only patterns in participants’ accounts, but also the underlying mechanisms through which entrepreneurial calling is experienced, particularly in relation to the interplay between meaning, autonomy, and psychological strain.

### 2.2 Research procedure

Initial contact was made via email using an institutional research email address associated with the study. Participants who agreed to take part received a written information sheet, an informed consent form, and a data management statement. Written consent was obtained prior to scheduling the interview. All interviews were conducted online via Zoom. At the start of each interview, participants provided verbal consent for audio recording. To ensure confidentiality, participants were assigned numerical identifiers (e.g., P1, P2), and no identifying information was used in transcripts or publications. Participants were informed of their right to withdraw from the study or decline to answer specific questions at any time without consequence.

Audio recordings were stored in a password-protected folder on a secure computer and transcribed verbatim for analysis. Transcripts were stored securely, and participants retained the right to request deletion of their data at any stage of the research process.

### 2.3 Participants

The sample consisted of 31 Hungarian entrepreneurs over the age of 18, including 16 women and 15 men. All participants self-identified their work as a calling, and the sample included both self-employed individuals and business owners, all of whom engaged in entrepreneurial activities involving self-directed work. While the sample was homogeneous in terms of entrepreneurial status, it was professionally diverse, encompassing activities such as chocolate making, stair manufacturing, language teaching, and other entrepreneurial domains (see Table 1). Age was not collected as it was not central to the research focus.

**Table 1:** Participant characteristics (*N* = 31).

Participant	Gender	Relationship and family status	Occupation
Participant 1	Female	In a relationship (no children)	Make-up artist
Participant 2	Male	Married with children	Ski instructor
Participant 3	Female	Married with children	Language teacher
Participant 4	Female	In a relationship (no children)	Lawyer, mediator
Participant 5	Female	Single (no children)	Innovative healthcare professional
Participant 6	Female	Married with children	Community space developer
Participant 7	Female	Single with children	Yoga instructor
Participant 8	Female	Married with children	Positive psychology coach
Participant 9	Male	Married with children	Staircase maker
Participant 10	Male	In a relationship (no children)	Lighting design and electrical systems specialist
Participant 11	Male	Married with children	Laser manufacturing and printing specialist
Participant 12	Female	Married (no children)	Kindergarten director
Participant 13	Male	Single (no children)	Grant writer
Participant 14	Female	Single (no children)	Restaurant owner
Participant 15	Male	Married with children	Product developer and manufacturer
Participant 16	Female	Single with children	Nail artist
Participant 17	Female	Married with children	Helping practitioner and trainer
Participant 18	Male	In a relationship (no children)	Startup CEO
Participant 19	Male	In a relationship (no children)	3D designer and prototype developer
Participant 20	Female	In a relationship (no children)	Chocolate maker and instructor
Participant 21	Female	Single with children	Wedding decorator
Participant 22	Female	Single (no children)	Artist
Participant 23	Male	Single (no children)	Design and branding agency founder
Participant 24	Male	Married with children	Business, leadership, and health coach
Participant 25	Male	Married (no children)	Agricultural business owner
Participant 26	Male	Married with children	Coffee shop owner
Participant 27	Male	Married with children	Journalist, TV reporter
Participant 28	Male	In a relationship (no children)	Construction entrepreneur
Participant 29	Male	Married (no children)	Ice cream shop owner
Participant 30	Female	Single (no children)	Business and marketing consultant
Participant 31	Female	Married with children	Singer and songwriter

“Children” refers to dependent children living in the household or requiring ongoing care. Participants with adult children living independently are categorized as “no children”.

Given the conceptual breadth of entrepreneurial calling and the diversity of work arrangements within entrepreneurship, a relatively large qualitative sample was sought to capture variation in lived experiences across gender, professional background, and life stage. This sample size enabled analytic depth while allowing comparison across diverse entrepreneurial pathways.

Participants were recruited through professional networks and targeted outreach to individuals engaged in self-directed entrepreneurial activities. Inclusion criteria required that participants explicitly described their work as a calling during initial contact or screening, ensuring a focus on individuals who actively interpret their work through this lens. This supports the study’s aim to explore how entrepreneurial calling is experienced in practice, while also shaping the types of experiences and interpretations captured in the data. At the same time, this sampling approach involves self-selection bias, as those who chose to participate may be more likely to frame and articulate their work in terms of calling. As a result, the findings reflect the experiences of individuals who actively construct their work as meaningful and identity-relevant, rather than representing the full range of entrepreneurial experiences. This has implications for transferability, as the study prioritizes depth of understanding within a specific interpretive group over representational breadth across all entrepreneurs.

### 2.4 Data analysis

We employed thematic analysis following the six-phase approach outlined by Braun and Clarke (2006). This method provides a flexible yet systematic framework for identifying patterns of meaning within qualitative data, and is well suited to exploring identity, meaning-making, and lived experience. The analysis was conducted inductively. Unlike grounded theory or content analysis, thematic analysis allows interpretive engagement with the data without needing to generate a formal theory or rely on frequency-based coding (Clarke and Braun, 2013).

The analysis followed an iterative and recursive process:

1. Familiarization with the data: all interviews were transcribed verbatim and read multiple times to ensure immersion. Initial impressions and recurring ideas were documented.
2. Generating initial codes: using an inductive approach, meaningful segments of data were coded manually, primarily at the semantic level, with attention to underlying psychological meanings where relevant.
3. Searching for themes: codes were clustered into broader candidate themes reflecting key aspects of entrepreneurial calling.
4. Reviewing themes: themes were refined through constant comparison to ensure internal coherence, distinctiveness, and empirical support. Some themes were merged, while others were divided into subthemes.
5. Defining and naming themes: themes were clearly defined to capture their core meanings and psychological, professional, and social dimensions.
6. Producing the report: themes were synthesized into a coherent narrative and illustrated with verbatim participant quotations.

The study focused on how participants themselves interpret and articulate calling. Each analytic step informed the next in an iterative process: initial coding provided the basis for identifying broader patterns, which were then refined into themes capturing recurring configurations of meaning across the dataset. The outcome was not only a set of descriptive themes, but a structured interpretive synthesis of how entrepreneurial calling is experienced, enacted, and sustained under conditions of autonomy, uncertainty, and psychological demand. The analysis focused on identifying broader patterns through which participants made sense of calling.

To enhance analytic credibility, the analysis involved repeated return to the original transcripts to ensure that emerging themes remained grounded in participants’ accounts. Coding and theme development were conducted iteratively across the dataset, with careful comparison between interviews to identify patterns of shared meaning while preserving nuance. Illustrative quotations are provided to maintain transparency between data and interpretation.

We also examined how different aspects of entrepreneurial calling were meaningfully related. Themes such as autonomy, strain, and growth were derived through an iterative process of comparing coded segments across interviews, identifying recurring patterns, and examining how these patterns co-occurred across interviews. An illustrative example of selected analytic steps is provided in the Supplementary material. The final themes represent analytically constructed patterns that capture how participants experienced and negotiated entrepreneurial calling. Each theme reflects not only recurring content within the data, but also a distinct aspect of how calling was enacted and psychologically sustained in everyday practice.

### 2.5 Translation of interview quotations

Interviews were conducted in Hungarian, and the initial coding and thematic analysis were carried out using the original Hungarian transcripts. Following theme development, quotations selected for publication were translated into English by the first author. The translations were reviewed by the second author and an academic writing specialist to ensure conceptual accuracy, clarity, and faithfulness to the tone of the original expressions. Where necessary, wording was refined iteratively to preserve participants’ intended meanings while maintaining readability in English.

### 2.6 Analytical rigor and trustworthiness

Analytical rigor was supported through attention to coherence, transparency, and interpretive depth. Rather than treating themes as isolated categories, we focused on developing an internally consistent interpretation of how different aspects of entrepreneurial calling were meaningfully related within participants’ accounts. Particular attention was given to preserving the complexity and ambivalence of participants’ experiences, especially in relation to the coexistence of meaningful engagement and psychological strain. This involved maintaining sensitivity to both shared patterns and variations across accounts, and avoiding premature simplification of the data. The use of illustrative quotations was intended not only to support interpretation but also to allow readers to engage directly with the underlying data.

In line with interpretive qualitative approaches, the aim of the analysis was to provide a theoretically informed and contextually grounded understanding of entrepreneurial calling as a lived work experience, in line with interpretive perspectives that emphasize understanding meaning as embedded in lived experience rather than as objective measurement (Sandberg, 2005), and with broader principles of qualitative inquiry (Kvale, 1996).

## 3 Results

The analysis identified four interconnected patterns through which participants enacted and sustained entrepreneurial calling in everyday work life. Accounts suggest that entrepreneurial calling operated as a dynamic and psychologically demanding process shaped through the ongoing negotiation of meaning, identity, autonomy, uncertainty, and self-regulation. The themes presented below capture these interconnected patterns and show how entrepreneurial calling was sustained.

### 3.1 Theme 1: calling as identity-congruent meaningful engagement

Most participants experienced their entrepreneurial work as extending beyond instrumental goals such as income generation or career advancement. Rather than describing work as a temporary occupational role, they framed it as deeply meaningful and closely intertwined with personal identity. Across accounts, meaning, identity, and engagement emerged as mutually reinforcing dimensions that together formed the experiential core of entrepreneurial calling. This can be understood as reflecting a mode of work engagement in which entrepreneurial activity becomes integrated into participants’ broader sense of self and purpose.

A recurring feature was intrinsic motivation rooted in creative engagement and an inner orientation toward the activity itself. Participants emphasized that their motivation stemmed less from external recognition or reward than from a sense of alignment, intuition, and authenticity: as one said, “One must listen to the feelings that arise.” (P7). Others spoke of being guided by intuition and a perceived relevance of their work for both themselves and others, for example: “Intuition and gut feeling are very important in recognizing what may be interesting and meaningful both to me and to others.” (P27). In describing this motivation, participants often framed entrepreneurial ideas as emerging from an inner source closely linked to authenticity, as in “An idea that springs from your innermost core, allowing you to live your most authentic life.” (P31).

What becomes apparent is that participants did not experience entrepreneurial work merely as enjoyable or stimulating, but as personally expressive and identity-congruent. This may imply that entrepreneurial calling involves a form of engagement in which work is experienced as aligned with one’s perceived “true self,” rather than simply as an externally rewarding activity.

This intrinsic orientation toward work was often accompanied by deep engagement and absorption. Participants described losing track of time, becoming fully immersed in their activities, and experiencing strong mental and emotional involvement, illustrated by comments such as “Well, it’s that flow experience that I can perhaps compare to being in love.” (P1), and “When you are doing your calling, time just flows.” (P11). They further described these experiences not as exceptional moments, but as embedded within everyday work life. Work was not framed in terms of a simple contrast between effort and enjoyment, but rather as worthwhile in itself, even when demanding or intensive. This is reflected in statements such as “The joy of everyday life - doing your work with joy feels like not working at all.” (P12) and “Work is not work for me. It is where I find fulfilment.” (P17).

They thus seem to experience their work as intrinsically meaningful in a sustained and ongoing manner, rather than temporarily motivating, implying enduring engagement where effort, fulfilment, and self-expression become closely interconnected.

As engagement deepened, participants increasingly described their work in terms of authenticity and alignment with personal values. Several contrasted their current entrepreneurial activities with earlier work experiences in which they had felt constrained or disconnected from themselves. One said, for example, “Now I can finally be myself.” (P22). Others referred to calling as involving self-understanding and identity exploration: “You can find your calling by first figuring out what you love to do and who you are.” (P30). Authenticity was also linked to interpersonal relationships, as reflected in “The quality of human relationships changes when we exist as our true selves.” (P31).

Alongside authenticity, participants frequently experienced work as meaningful when it involved helping, teaching, or contributing to others. Such experiences were described as integral to the value and significance of their work, reflected in statements such as “I feel like I’m doing something for humanity.” (P4) and “It feels good to know that I can help people.” (P13). This suggests they often constructed entrepreneurial calling not solely in individualistic terms, but also in relation to perceived contribution, usefulness, and social significance.

Strong engagement with work was also reflected in a distinctive temporal relationship to work. Several expressed eagerness to return to work after weekends or holidays rather than perceiving it as something to be endured until periods of rest, as illustrated by comments such as “I couldn’t wait for the weekend to end so I could get back to work.” (P19) and “I have no problem with Monday mornings.” (P30). They frequently described work as inseparable from their sense of self: “This is not only my calling, but also my love.” (P16), “They say that I am the brand of (name of the company anonymized).” (P6), and “My personality and my work are completely intertwined.” (P21).

These accounts, then, reflect a particularly strong integration between work and identity, in which entrepreneurial activity becomes closely connected to participants’ self-concept and everyday experience of meaning. Importantly, this identity-based engagement appears to distinguish entrepreneurial calling from forms of entrepreneurial motivation rooted primarily in achievement, financial reward, or autonomy alone.

Calling thus emerged as an experience in which meaning, motivation, engagement, and identity were tightly intertwined in everyday work life. Work was not just seen as meaningful; rather, calling was constructed as an identity-based mode of engagement interconnecting self-concept, motivation, and daily activity. This alignment between self and work forms the experiential foundation of entrepreneurial calling and provides the basis for understanding how later themes, such as autonomy, responsibility, and strain, become intensified within entrepreneurial contexts.

All in all, then a pattern emerges where entrepreneurial calling was experienced as identity-congruent and intrinsically meaningful engagement through which work became closely integrated with participants’ sense of self, purpose, and everyday experience. This strong alignment also helps explain why later themes, such as autonomy, responsibility, and psychological strain, became especially salient within entrepreneurial contexts.

### 3.2 Theme 2: calling as autonomy-enabled enactment

While Theme 1 captures the experiential core of calling, these experiences are also embedded within specific structural conditions, notably autonomy. Work was seen as taking place within a context characterized by freedom and self-direction. Autonomy thus appeared not as part of the experience itself, but as a condition that enabled and shaped how calling was enacted in practice. Participants often experienced entrepreneurial activity as allowing them to organize work on their own terms, with autonomy being a recurring feature of how they described living their calling in everyday work life.

The importance of self-direction in shaping their daily work lives was repeatedly emphasized, particularly the chance to decide how, when, and with whom they worked. One said, “I work when I want, where I want, and with whom I want.” (P24). Others referred to having the freedom to refuse opportunities that did not align with their values, for example “I can say no to business opportunities that don’t align with my values.” (P30), and “I have never done a report that went against my soul.” (P27).

Time autonomy was also frequently linked to freedom at work. Participants described having flexibility over how they structured their days and responded to personal needs: “I have more freedom over my time; I decide how I spend my days.” (P9) and “If today I feel that it’s better for me to go running, I can do that.” (P18). What these accounts suggest is that autonomy was experienced not merely as schedule flexibility, but as the possibility of organizing work in ways perceived as congruent with personal needs, values, and rhythms of life.

Decision-making autonomy thus emerged as another recurrent aspect of participants’ accounts. Several described their entrepreneurial path in terms of distancing themselves from hierarchical control and external authority, often in contrast to earlier organizational experiences, as reflected in statements like “I didn’t want any more bosses. I had had enough.” (P10). Others emphasized independence and self-reliance, for example “It feels really good to stand on your own feet and not depend on others.” (P20). We see then a desire not only for independence itself, but for greater ownership over the relationship between work and self, with autonomy closely connected to the ability to sustain alignment between work activities and personal identity.

At the same time, participants also associated autonomy with responsibility and risk. Some described being accountable for others: “There are people who listen to me and for whom I am responsible.” (P25), while others contrasted entrepreneurial autonomy with the perceived security of organizational employment: “People are afraid to leave the multinational because they see security in it.” (P24). There was also mention of extended working hours and difficulty disengaging from work: “Even when I go on vacation, what’s happening at the company is always on my mind.” (P18).

From these accounts we see how autonomy was experienced simultaneously as enabling and demanding. It both allowed participants to organize work in personally meaningful and identity-congruent ways, yet intensified responsibility, involvement, and psychological investment in work outcomes. Autonomy therefore appeared to function not simply as freedom from external control, but as a mechanism through which entrepreneurial calling became more deeply integrated with participants’ identities and everyday lives. In this sense, the same autonomy that enabled meaningful self-direction also intensified emotional exposure, personal responsibility, and vulnerability to later-described experiences of strain, boundary blurring, and difficulty disengaging from work.

### 3.3 Theme 3: calling as psychologically demanding engagement

Accounts indicate that those features that made their work deeply involving and personally significant were also closely linked to strain, uncertainty, and difficulty disengaging. These challenges were frequently described not as external to work itself, but as emerging from participants’ personal involvement in it. The psychological demands participants described thus appear as inseparable from the meaningful and identity-relevant nature of their work.

Financial uncertainty was widely mentioned, with several referring to income unpredictability and fluctuating workloads, and describing the need to manage periods of intensive work followed by slower phases: “There are three months when I work around the clock and then there is much less work.” (P20). Others reflected more broadly on uncertainty as an enduring stressor inherent in entrepreneurial life. Some described the inability to anticipate future outcomes as psychologically demanding even when they strongly identified with their work, for example: “We cannot see into the future and this is an enormous stress factor for entrepreneurs.” (P13).

What becomes apparent here is that uncertainty was not experienced merely as an external business condition, but as something emotionally intensified through participants’ deep personal investment in their work. Entrepreneurial calling may thus amplify the psychological significance of uncertainty because work outcomes become closely connected to self-worth, identity, and perceived purpose.

Beyond financial concerns, numerous participants reported emotional exhaustion and psychological overload linked to the sustained intensity of calling-oriented work. Several mentioned feeling emotionally drained, where work sometimes displaced attention to their own personal needs: “My own emotional life has been pushed into the background.” (P5). Others described the cumulative impact of prolonged work demands more broadly, for example: “It completely wears you down. It was damn hard and I think it still is.” (P13).

Importantly, participants frequently described this strain as difficult to separate from their commitment to work itself. Because work was experienced as closely intertwined with identity and purpose, disengaging from work was often experienced not simply as reducing effort, but as distancing oneself from something personally meaningful. This suggests that the same identity-based engagement that generated meaning and fulfilment could also make psychological detachment from work more difficult. One long-established entrepreneur articulated this paradoxical intensity directly: “As much freedom as it gives, it also takes over your life.” (P26).

Blurred boundaries between work and personal life were also frequently mentioned. Participants referred to difficulties in setting limits around time, availability, and emotional investment, noting that boundary-setting often had to be learned over time, as illustrated by comments such as “I had to learn to set boundaries; otherwise, it would have taken everything.” (P8) and “It’s very difficult to say no and decide when to put down your laptop.” (P18). Several also commented on tensions between work commitments and personal relationships, particularly family life, with statements such as “Countless hours of work, time taken away from family.” (P26) and “My wife always complained that I was always in the workshop.” (P19).

The fact that such tensions were described across different relationship and family statuses suggests that difficulties with disengagement and boundary management were not limited to specific life situations alone, although they appeared particularly salient in relation to family responsibilities and emotional availability. This may suggest that entrepreneurial calling extends beyond a work role and increasingly shapes broader relational and everyday life contexts.

Changes in how participants talked about success, failure, and challenge over time were also visible. Rather than describing entrepreneurial experiences in terms of clear-cut success or failure, they increasingly framed challenges as part of evolving life situations requiring endurance and continued adjustment. These reflections were often articulated in relation to psychological perseverance: “I realized that there is no success or failure, only life situations.” (P5). Others referred to repeatedly starting again despite setbacks, for example “I have learned very well how to start over.” (P31). Some expressed attitudes that de-emphasized fear in relation to pursuing their work: “There is no room for fear when building your own castle.” (P15).

It appears, then, that experiences of strain, uncertainty, and boundary blurring therefore appeared closely connected to the meaningful and identity-relevant nature of entrepreneurial calling itself, rather than representing only general pressures associated with entrepreneurship. Entrepreneurial calling thus emerged as a psychologically demanding form of engagement requiring continuous adaptation and regulation across different work and life domains.

### 3.4 Theme 4: sustaining calling through self-regulation, growth, and adaptation

Participants widely discussed learning, resilience, and self-knowledge as processes through which challenges contributed to a deeper understanding of themselves, their work, and how to continue despite uncertainty. Several described engaging in continuous self-work alongside their entrepreneurial activities, referring to continuous reflection and personal development as integral to their everyday work experience. As one explained: “Through my work, I am constantly working on myself, which involves continuous self-reflection and conscious development.” (P17).

Learning was described as closely intertwined with the development of self-knowledge, particularly through confronting limits, difficulties, and mistakes. Narratives suggest that these learning processes were not separate from the challenges they experienced, but emerged through continuous efforts to manage uncertainty, responsibility, and sustained involvement in their work. In this sense, struggle itself appeared to function as a mechanism through which participants interpreted and negotiated their entrepreneurial calling over time. Participants referred to learning through difficulty as a way of better understanding their own capacities, preferences, and boundaries. For example: “We must learn to live with challenges.” (P21) and “Becoming a leader involved a lot of struggle but I solved it through learning.” (P12).

Participants also described developing greater self-knowledge through accumulated experience and experimentation: “I had tried so many things before that I knew exactly what I didn’t want to do.” (P28). This may suggest that entrepreneurial calling was not experienced as a fixed inner certainty from the outset, but as gradually clarified and refined through sustained engagement with work-related challenges.

Participants also mentioned persevering despite adversity and adjusting their approach over time. These experiences were frequently discussed in relation to resilience and adaptation, where some said, “Whatever challenge came our way, we sat down, thought it through, and moved on.” (P29), “So, we always had to make changes, but the goal was always there.” (P2), or “When something bad happens to me, I always think about how I can turn it into something good.” (P9). These reflections suggest that resilience was not understood simply as endurance or persistence, but as an active process of reinterpretation and adjustment through which participants attempted to sustain meaningful engagement despite continuous demands and uncertainty.

Growth was also reflected in perceived changes in personality, confidence, and self-perception over time. Participants described becoming more assertive, self-assured, or capable through their entrepreneurial experiences, as illustrated by “As a child, I was shy, but today I negotiate and give presentations with confidence.” (P11) and “They criticized my work, but I knew I had worked hard for it.” (P23). Such accounts may indicate that entrepreneurial calling was experienced not only as work engagement, but also as contributing to broader processes of identity development and personal transformation.

These accounts suggest that these entrepreneurs experienced growth not simply through success, but through ongoing engagement with uncertainty, responsibility, and psychological demands. Challenges were often framed as opportunities for learning, adaptation, and increased self-knowledge, indicating that resilience and self-regulation played an important role in sustaining entrepreneurial calling over time.

Participants also described entrepreneurial calling as something clarified gradually through experimentation, self-reflection, and accumulated experience rather than immediately recognized from the outset. As one explained: “People don’t always find their calling right away. It’s important that if you realize you’re not going in the right direction, you dare to change course.” (P12). Self-knowledge was similarly described as essential for sustaining entrepreneurial engagement: “First, you have to get to know yourself to see if you’re suited to this way of life.” (P18).

Some also emphasized the practical demands of sustaining entrepreneurial work over time, including financial awareness and long-term adjustment. As one explained: “I learned to build up reserves so that ups and downs would not ruin my business.” (P11). Entrepreneurial calling was thus sustained not through certainty or continuous positive experience, but through continuous self-reflection, adaptation, and psychological regulation across both personal and professional domains.

Across the four themes, entrepreneurial calling emerged as a psychologically demanding form of work engagement in which meaning, autonomy, uncertainty, and personal responsibility became closely intertwined over time. Rather than a stable positive state, calling was experienced as requiring continuous adaptation, boundary management, and self-regulation to sustain meaningful engagement under conditions of uncertainty.

## 4 Discussion

### 4.1 Entrepreneurial calling as a dynamic work experience and its implications for psychological wellbeing

This study sought to explore how entrepreneurs who perceive their work as a calling make sense of this experience in their everyday working lives, with particular attention to psychological wellbeing. Building on existing research (Dik et al., 2012; Bunderson and Thompson, 2009), our analysis suggests that calling is enacted, negotiated, and sustained through everyday work practices in entrepreneurial contexts, in line with Schou’s (2022) work on entrepreneurial calling as a continuous narrative and sensemaking process. However, our findings extend Schou’s perspective, showing how entrepreneurial calling is psychologically sustained and regulated in everyday work practice under conditions of autonomy, uncertainty, and ongoing responsibility.

Calling is thus revealed as an ongoing way of engaging with work through concrete activities such as creating, helping, teaching, and building. This aligns with work orientation perspectives that locate meaning in how work is experienced (Wrzesniewski et al., 1997; Dik and Duffy, 2009), suggesting it depends less on one’s job type and more on how one experiences and approaches work.

Crucially, entrepreneurship did not emerge as the object of calling, but rather as a work arrangement through which calling could be enacted. Entrepreneurial settings appeared to enable a close alignment between work, identity, and values, while simultaneously exposing individuals to uncertainty, responsibility, and psychological strain. Calling therefore emerged as a dynamic work experience made possible by entrepreneurship, one that simultaneously functioned as a source of meaning, purpose, and identity integration while also intensifying psychological exposure, emotional strain, and difficulties with disengagement from work.

**Figure 1:**
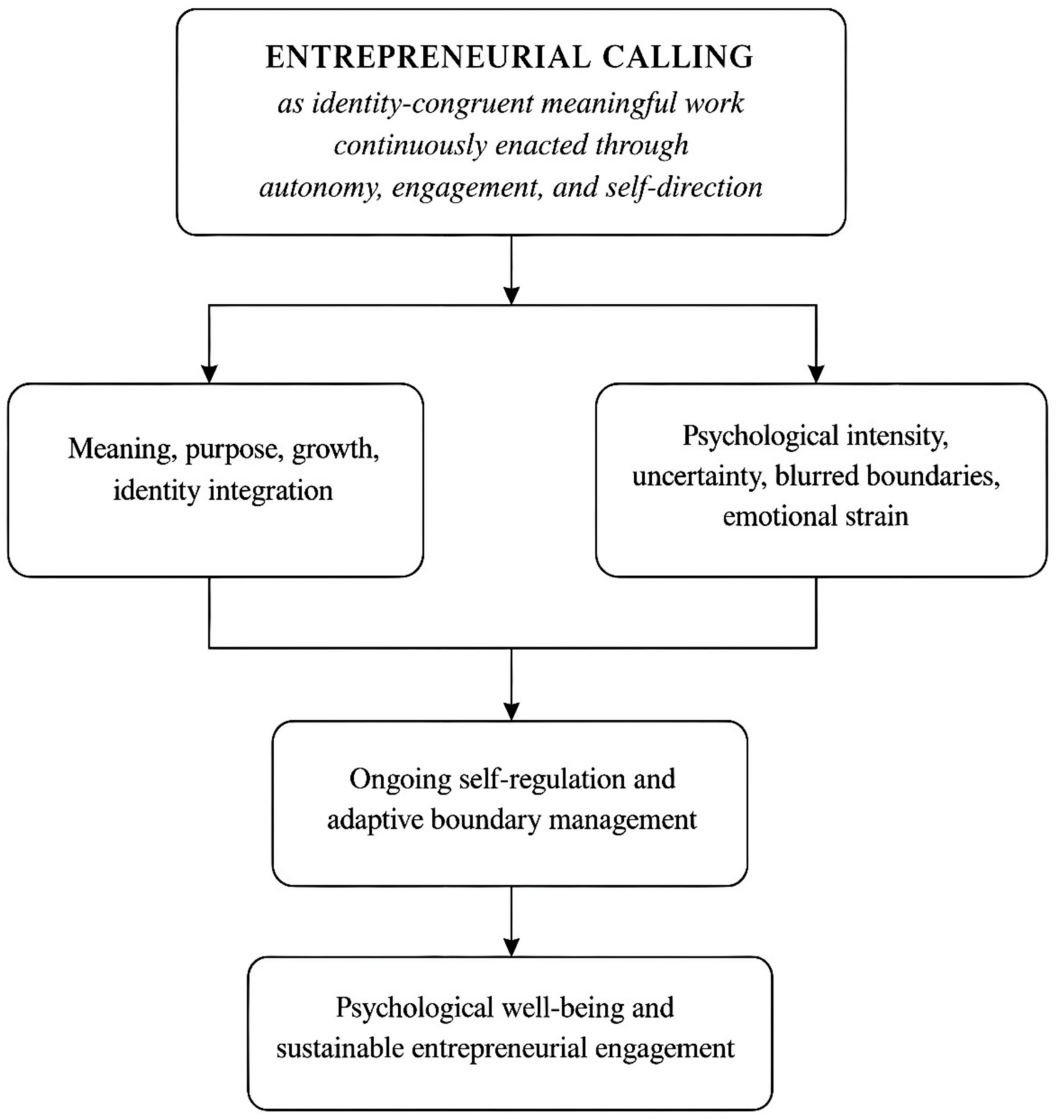
A dynamic regulatory model of entrepreneurial calling and psychological wellbeing.

Overall, the findings suggest that entrepreneurial calling is sustained through a dynamic interplay between identity-relevant engagement, autonomy, uncertainty, and continuous self-regulation. This extends predominantly static conceptualizations of calling by showing how entrepreneurial calling is continuously negotiated within entrepreneurial work contexts. To integrate these dynamics, Figure 1 presents a conceptual model illustrating how entrepreneurial calling is psychologically sustained within entrepreneurial work contexts.

### 4.2 Meaningful engagement and psychological sustainability

Narratives reveal how calling is experienced as a sustained form of engagement integrating effort, enjoyment, and commitment over time. Work was not framed as something to be balanced against life, but as a central domain through which identity, direction, and coherence were maintained. This resonates with research linking calling to intrinsic motivation and identity integration (Bunderson and Thompson, 2009; Dobrow and Tosti-Kharas, 2011), yet our findings also suggest that strong integration between work and identity does not automatically translate into psychological wellbeing. Instead, we see a more ambivalent process where meaningful engagement simultaneously functions as a source of fulfilment and heightened psychological exposure.

Psychological wellbeing emerged not as a stable outcome of meaningful work, but as a dynamic process shaped by how meaning and identity were sustained under conditions of uncertainty and responsibility. While meaningful engagement was described as energizing and purposeful, it also amplified vulnerability when disengagement became difficult. These findings confirm the ambivalence of calling (Duffy and Dik, 2013; Thompson and Bunderson, 2019) but also show how this ambivalence unfolds through sustained work engagement rather than as an abstract psychological tension.

In this regard, we also extend Schou’s (2022) narrative perspective on entrepreneurial calling by showing how entrepreneurial sensemaking is closely intertwined with psychological regulation. Participants did not merely describe calling as meaningful work, but as an ongoing process of negotiating engagement, identity, strain, and recovery over time. This aligns with second wave positive psychology perspectives that see wellbeing as effortful, developmental, and embedded in engagement with meaningful but challenging life domains (Lomas and Ivtzan, 2016; Wong et al., 2022). This suggests that wellbeing in entrepreneurial calling is sustained not through the absence of difficulty, but through individuals’ capacity to remain engaged with meaningful work while simultaneously regulating its psychological costs. Psychological wellbeing therefore appeared closely tied to participants’ capacity to manage engagement intensity, recovery, and personal limits over time.

These dynamics also reflect multiple dimensions of eudaimonic wellbeing, particularly purpose in life, autonomy, and self-acceptance, which appeared to evolve through sustained engagement in entrepreneurial work (Shir and Ryff, 2021). Calling-related wellbeing depended on ongoing processes of regulation and adaptation under sustained psychological demands.

### 4.3 Autonomy as an enabling but demanding condition of calling

A central contribution of this study concerns the role of autonomy in shaping how calling is lived within entrepreneurial contexts. Autonomy was not experienced as a peripheral benefit, but as a foundational condition enabling participants to align their work with personal rhythms, values, and perceived needs. This finding echoes eudaimonic perspectives on psychological wellbeing, which identify autonomy as an important dimension of meaningful and self-directed functioning (Ryff, 1989; Ryff and Keyes, 1995), as well as entrepreneurship research highlighting autonomy as a key motivational driver (Stephan, 2018). It also aligns with research suggesting that entrepreneurial activity allows individuals to actively shape their work, identity, and everyday way of living over time (Shir and Ryff, 2021).

From this perspective, autonomy is not just a contextual feature of entrepreneurship, but a psychological mechanism through which entrepreneurial calling becomes enactable in everyday work practice. This helps explain why entrepreneurial calling can simultaneously intensify both psychological fulfilment and psychological demand.

Participants’ descriptions of actively structuring their schedules, regulating workload intensity, and making decisions congruent with their evolving sense of self illustrate how autonomy functioned as a form of environmental mastery—the capacity to shape one’s work conditions in alignment with personal needs and limits. This did not so much reduce psychological strain as transforming its meaning. Responsibility and risk became internalized as self-chosen conditions of work. Challenges were more readily tolerated when experienced as self-endorsed, consistent with research suggesting that autonomy strengthens psychological ownership over work outcomes (Cardon and Kirk, 2015). At the same time, this autonomy relocated responsibility for regulation, boundaries, and sustainability largely onto the individual. The same autonomy that enabled meaningful self-direction also intensified responsibility for maintaining personal boundaries and psychological sustainability over time.

### 4.4 Psychological intensity, strain, and self-regulation

While prior research has documented general stressors in entrepreneurship (Ahmed et al., 2022; Stephan, 2018), our findings show these challenges take on a distinct quality when work is experienced as a calling, becoming closely tied to identity, meaning, and sustained engagement.

Accounts revealed substantial psychological and relational costs associated with living a calling in entrepreneurial contexts. Financial uncertainty, fluctuating workloads, and sustained responsibility for outcomes were described as enduring features of their work lives. Because calling was experienced as closely intertwined with identity, work-related demands proved difficult to contain psychologically, contributing to emotional depletion and difficulty disengaging from work. These patterns align with research on work devotion and identity-based commitment, where deep investment can intensify strain rather than buffer against it (Bunderson and Thompson, 2009).

Importantly, difficulty and strain were not interpreted as evidence that calling had lost its meaning. Rather, they were often understood as arising precisely because the work mattered deeply, reinforcing the identity-based nature of calling described in prior research (Dobrow et al., 2023). Strain therefore coexisted with purpose, rather than negating it.

Boundary-blurring between work and personal life further amplified these costs, particularly in relational and helping-oriented work where emotional availability extended beyond formal working hours. Calling thus reshaped participants’ relational worlds— sometimes deepening connection, while at other times constraining emotional presence in private life. These tensions were particularly pronounced in the context of relational commitments (see Table 1), suggesting that the demands of calling may be experienced differently depending on individuals’ life circumstances.

We see, then, that entrepreneurial calling is not best understood as an inherently beneficial or uniformly positive work experience. Rather, it intensified psychological exposure by making work-related uncertainty, responsibility, and demands more personally consequential, emotionally salient, and difficult to disengage from, as work outcomes became closely intertwined with identity, self-worth, and perceived purpose. This further underscores the central role of regulation in sustaining long-term psychological wellbeing.

### 4.5 Growth, resilience, and wellbeing as emergent processes

Despite the psychological demands described above, participants’ narratives also suggest that entrepreneurial calling functioned as a developmental context through which learning, resilience, and self-knowledge gradually emerged. These patterns resonate with narrative perspectives on calling, which conceptualize calling as an ongoing sensemaking process through which individuals construct and reconstruct the meaning of their work and identity over time (Bloom et al., 2021).

Sustaining engagement with meaningful work was portrayed as requiring continuous self-work, including reflection, emotional regulation, and the recalibration of expectations over time. This aligns with eudaimonic models of psychological wellbeing that emphasize personal growth and self-acceptance as dynamic and effortful dimensions of functioning (Ryff and Keyes, 1995). Importantly, growth did not appear as a deliberate strategy to enhance wellbeing, but as an emergent outcome of remaining engaged with work that was both meaningful and demanding.

In this context, resilience was described less as a stable personality trait and more as a capacity developed through experience. Participants reported learning to reinterpret setbacks, adjust aspirations, and persist despite fatigue or uncertainty, in line with conceptualizations of resilience as a dynamic and contextually shaped process that emerges through continued engagement with meaningful but psychologically demanding work (Stephan, 2018; Uy et al., 2013). Self-knowledge played a central regulatory role, as they increasingly recognized both their limits and their strengths. This growing self-awareness was central in sustaining wellbeing while remaining committed to calling-oriented work.

### 4.6 Calling as a dynamic work practice

Entrepreneurial calling emerged as a sustained and dynamically negotiated work practice rather than as a stable identity label or purely motivational orientation. In line with Schou’s (2022) narrative conceptualization of entrepreneurial calling, participants described calling as something enacted and interpreted through everyday work activities. However, our findings demonstrate that this process is not only identity-related and meaning-oriented, but also psychologically regulatory, requiring individuals to negotiate autonomy, engagement, strain, and recovery over time.

Viewing entrepreneurial calling in this way offers a more nuanced understanding of meaningful work as a developmental, effortful, and psychologically complex experience rather than a uniformly beneficial state.

### 4.7 The dynamics of entrepreneurial calling and psychological wellbeing

Drawing on Bloom et al.’s (2021) process-oriented perspective on calling and narrative identity construction, the model in Figure 1 conceptualizes entrepreneurial calling as a dynamic regulatory process. Rather than treating calling as a relatively stable motivational orientation, the model emphasizes the ongoing psychological processes through which meaningful and identity-relevant entrepreneurial work is enacted, negotiated, and sustained under conditions of autonomy, uncertainty, and psychological demand.

More specifically, the model illustrates how autonomy and identity-congruent engagement simultaneously generate psychological resources, such as meaning, authenticity, and personal growth, while also intensifying emotional strain, uncertainty, over-engagement, and boundary challenges. Psychological sustainability therefore depends on continuous processes of self-regulation, adaptation, recovery, and boundary management across both work and personal life domains.

In this way, the model integrates narrative, eudaimonic, and entrepreneurial perspectives into a dynamic framework of entrepreneurial calling and psychological wellbeing.

### 4.8 Implications for practice and future research

Future research on calling might focus on how it is sustained in everyday work life as a fluctuating orientation. Longitudinal and qualitative approaches may be particularly useful in examining how psychological consequences evolve over time. For practitioners working with entrepreneurs, the findings highlight the importance of supporting sustainable engagement alongside deeply meaningful and identity-relevant work. When work is deeply meaningful and identity-defining, reflective capacity, boundary management, and self-regulation become critical for maintaining psychological wellbeing. Interventions that encourage calling should therefore also address its potential psychological costs. Future studies could further examine when and for whom entrepreneurial calling supports long-term wellbeing, and under what conditions it may increase vulnerability.

### 4.9 Study limitations

This study is exploratory and based on qualitative interviews with entrepreneurs who self-identified their work as a calling. The results are not intended to be generalizable, but to provide in-depth insight into how calling is experienced and interpreted. The analysis relies on retrospective self-reports, reflecting participants’ meaning-making rather than real-time behavioral observation. The study is situated within a Hungarian cultural and economic context, and the patterns described may reflect context-specific features of entrepreneurship and work values. Future research could include entrepreneurs who do not identify their work as a calling in order to further examine differences in meaning-making, motivation, and psychological wellbeing across entrepreneurial experiences.

## 5 Conclusion

Our examination of entrepreneurial calling as a dynamically enacted and psychologically negotiated form of meaningful work engagement within entrepreneurial contexts shows how it is sustained not simply through passion or motivation, but through ongoing processes of self-regulation, adaptation, and psychological negotiation under conditions of uncertainty. Rather than being a uniformly positive or self-sustaining source of wellbeing, it emerged as an evolving and psychologically complex work experience whereby meaning, identity, autonomy, strain, and personal growth became closely intertwined over time. The study thus contributes to a more process-oriented understanding of meaningful work, entrepreneurial wellbeing, and calling as lived and dynamically negotiated experience.

## Data Availability

The datasets presented in this article are not readily available because the qualitative data supporting the findings of this study consist of in-depth interview transcripts and audio recordings, which are not publicly available due to ethical considerations, participant confidentiality, and the sensitive nature of personal narratives. The interview questions and an example of the analytic process are provided in the Supplementary material to enhance transparency. Requests to access the datasets should be directed to jakab.judit@ppk.elte.hu.
