# Supplementary material for: Entrepreneurial calling and psychological wellbeing: a qualitative study of a dynamic and regulated work experience
Source: Front Psychol. 2026 Jun 24;17:1830938. doi: 10.3389/fpsyg.2026.1830938 (PMC13341500; doi:10.3389/fpsyg.2026.1830938)

Supplementary Material

1 Interview questions translated from Hungarian

Original Hungarian	English translation
1. Kérem, mondja el, mivel foglalkozik, mi a hivatása?<br><br>2. Kérem, fejtse ki, milyen érzés nap mint nap ezt a munkát végezni? Mi motiválja?<br><br>3. Mikor érzett először elhívást, vonzódást mostani munkájához és hogyan indult el? Kérem, beszéljen a konkrét fordulópontról, amennyiben volt ilyen. (Itt lehet kérni összehasonlítást a hivatás előtti és utáni életről.)<br><br>4. Milyen akadályokkal, kihívásokkal szembesült amióta hivatását űzi, és hogyan győzte le azokat? Kérem, térjen ki az átlendüléshez szükséges jellemvonásokra is.	1. Please tell me about your profession. What is your calling?<br><br>2. Please describe what it feels like to do this work every day. What motivates you?<br><br>3. When did you first feel drawn to your current work and how did you get started? Please talk about any specific turning points if there were any. (You may compare your life before and after discovering your calling.)<br><br>4. What obstacles and challenges have you faced since pursuing your calling, and how did you overcome them? Please elaborate on the personal qualities needed to navigate these difficulties.

5. Az, hogy a hivatásának él, mennyire határozza meg az Ön személyiségét?<br><br>6. Milyen lenne az élete a hivatása nélkül?<br><br>7. Milyen előnyökkel jár a munkája? Gondoljon magára, a családjára, ügyfeleire és a környezetére.<br><br>8. Milyen lemondásokkal, hátrányokkal jár a munkája?<br><br>9. Kérem, mondjon három dolgot, amiért érdemes belevágni egy hivatásból végzett karrierbe?<br><br>10. Ha adhatna tanácsot annak, aki még nem a hivatásában dolgozik, de szeretne erre az útra lépni, mi lenne az?	5. To what extent does living your calling shape your personality?<br><br>6. What would your life be like without your calling?<br><br>7. What are the advantages of your work? Consider yourself, your family, your clients, and your broader environment.<br><br>8. What sacrifices or disadvantages come with your work?<br><br>9. Please name three reasons why it is worth pursuing a career based on one's calling.<br><br>10. If you could give advice to someone who is not yet working in their calling but would like to pursue it, what would it be?

2 Illustrative example of the thematic analysis process

Participant 3 (P3)

This appendix presents an illustrative example of interview-level analytic development to enhance transparency regarding the thematic analysis process. It demonstrates how segments of text were interpreted and provisionally organized prior to cross-case theme construction. As outlined in the Data Analysis section, themes were developed iteratively across the full dataset; therefore, this example reflects analytic engagement with one interview rather than the final thematic structure.

2.1 Data familiarization

This interview explores the process of finding a calling, the challenges and benefits of entrepreneurship, and the pursuit of self-actualization. The participant reflects on fundamental moments in the journey, describing the transition from office work to full-time Italian language teaching, interpreting, and organizing cultural programs. The advantages of entrepreneurship such as freedom, creativity, and authenticity are emphasized, while the downsides, including loneliness, lack of feedback, and difficulties in setting boundaries, are also acknowledged.

2.2 Initial coding (Identifying key concepts)

An inductive coding approach was employed. Meaningful segments were coded primarily at the semantic level, while noting where narratives suggested broader psychological processes (e.g., identity affirmation, boundary negotiation). Coding was iterative, with labels refined as the analysis progressed. The table below presents examples of initial codes and corresponding data excerpts.

Code	Example from the interview

**I don't perceive it as work**	"I don't experience this as a job."
**Immediate and tangible results are motivating**	"I am extremely motivated by the fact that this job provides tangible and almost immediate results."
**Turning point: Transition to entrepreneurship**	"I drank two shots of brandy and wrote down the line 'I quit' and sent it off."
**Accidental beginnings**	"One day, I got a call asking me to help because their Italian teacher was unavailable."
**Solitude in entrepreneurship**	"There is no team. There is no one to relate to, no feedback."
**Difficulties receiving feedback**	"I always praise my students, but I rarely receive praise myself."
**Challenges in setting boundaries**	"I let people see into my life and it didn't end well."
**Accepting financial uncertainty**	"Now I tell myself that if there's a day without income, then fine, there's no money that day."
**Authenticity in calling**	"Since then, I can truly say that I am who I am."
**Importance of freedom**	"Experiencing this level of freedom in today's world is a huge privilege."

**No perfect moment to start a business**	"You shouldn't wait for the right moment. Just start and see what happens."

2.3 Grouping codes into preliminary patterns

At this stage, related codes were clustered to explore conceptual coherence within the interview. These preliminary patterns supported later cross-case comparison and refinement into shared themes across participants.

Theme	Related Codes	Explanation
**Connection between calling and authenticity**	I don't perceive it as work, Authenticity in calling	The participant believes that true calling does not feel like work but serves as a source of joy.
**Transition to entrepreneurship**	Turning point: Transition to entrepreneurship, Accidental beginnings	Although the journey began through spontaneous events, it was followed by a conscious decision.
**Challenges of entrepreneurship**	Solitude in entrepreneurship, Difficulties receiving feedback, Challenges in setting boundaries, Accepting financial uncertainty	While entrepreneurship provides freedom, it lacks external support and stability.

**Importance of freedom and creativity**	Importance of freedom, Immediate and tangible results are motivating	Creativity and flexibility are essential components of their work.
**Necessity of starting a business**	No perfect moment to start a business	The participant believes there is no perfect time to start - one must take action.

2.4 Illustrative quotations related to preliminary patterns

Theme	Related quote
**Connection between calling and authenticity**	"Since then, I can truly say that I am who I am."
**Transition to entrepreneurship**	"I drank two shots of brandy and wrote down the line 'I quit' and sent it off."
**Challenges of entrepreneurship**	"There is no team. There is no one to relate to, no feedback."
**Importance of freedom and creativity**	"Experiencing this level of freedom in today's world is a huge privilege."

**Necessity of starting a business**	"You shouldn't wait for the right moment. Just start and see what happens."

2.5 Interview-level analytic summary

For this participant, entrepreneurship is constructed not merely as a professional pathway but as an existential way of living that enables freedom, creativity, and authenticity. Work is not experienced as "work" in the conventional sense; rather, it is narrated as a source of intrinsic joy and sustained motivation. The transition into entrepreneurship is framed as both contingent and agentic - shaped by unexpected opportunities yet solidified through deliberate and courageous choice. At the same time, the narrative does not idealize this pathway. Experiences of loneliness, absence of feedback, blurred boundaries, and financial uncertainty are acknowledged as integral tensions within the entrepreneurial journey. Well-being appears to emerge not from the mere presence of calling, but from the gradual negotiation of these tensions and the development of psychological balance over time. A central meaning-making element in the account is the rejection of the idea of a "perfect moment." Instead, commitment, courage, patience, and perseverance are positioned as necessary conditions for sustaining a calling-driven life. The narrative thus frames entrepreneurial engagement as an ongoing developmental process in which stability is not given at the outset but cultivated through continued participation and trust in the unfolding journey.

3 Graphical abstract

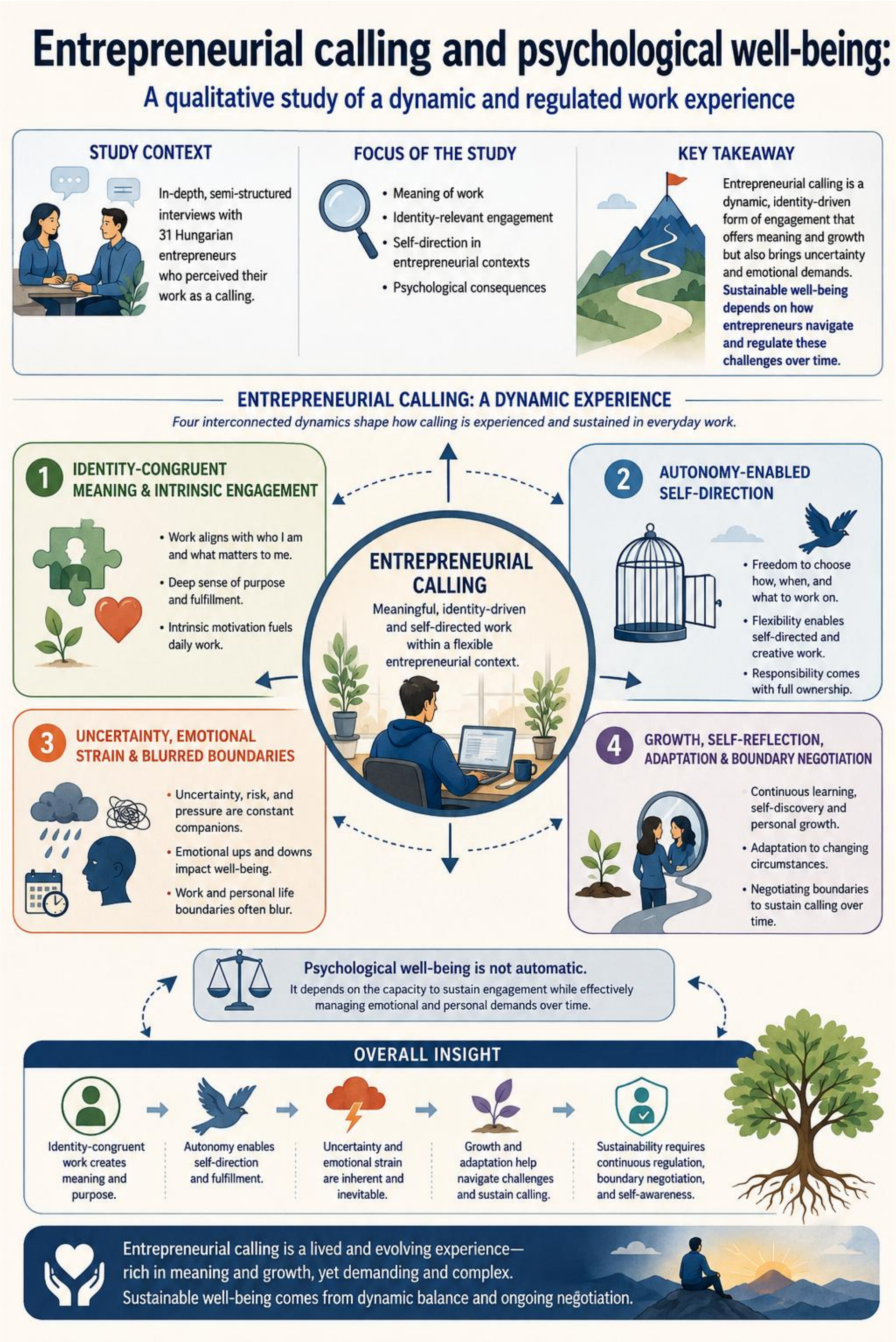

4 Dynamic Regulatory Model of Entrepreneurial Calling and Psychological Well-Being

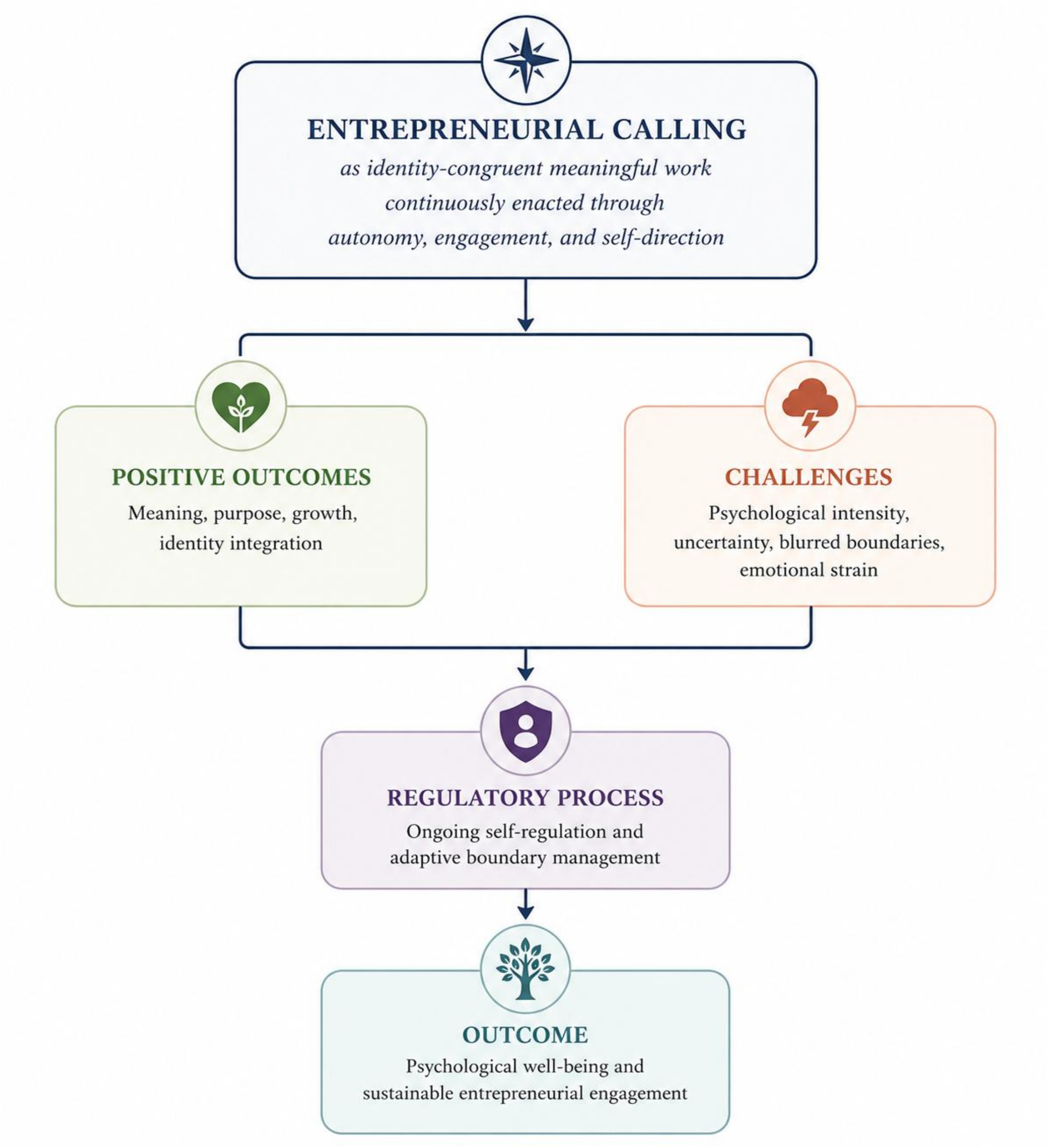

Supplement: Supplementary file 1 [file Supplementary_file_1.pdf]
